# How executive functions predict development in syntactic complexity of narrative writing in the upper elementary grades

**DOI:** 10.1007/s11145-016-9670-8

**Published:** 2016-07-01

**Authors:** Elise Drijbooms, Margriet A. Groen, Ludo Verhoeven

**Affiliations:** Behavioural Science Institute, Radboud University, Montessorilaan 3, P.O. Box 9104, 6500 HE Nijmegen, The Netherlands

**Keywords:** Executive functions, Narratives, Longitudinal study, Syntactic complexity, Writing development

## Abstract

The aim of this study was to examine the contribution of transcription skills, oral language skills, and executive functions to growth in narrative writing between fourth and sixth grade. While text length and story content of narratives did not increase with age, syntactic complexity of narratives showed a clear developmental progression. Results from path analyses revealed that later syntactic complexity of narrative writing was, in addition to initial syntactic complexity, predicted by oral grammar, inhibition, and planning. These results are discussed in light of the changes that characterize writing development in the upper elementary grades. More specifically, this study emphasizes the relevance of syntactic complexity as a developmental marker as well as the importance of executive functions for later writing development.

## Introduction

Learning to write is an essential, yet challenging, part of literacy acquisition in the elementary grades that is supported by a number of important component skills. According to a pivotal, developmental model of writing (Berninger & Winn, 2006), three interrelated component skills underlie written text production, as they interact functionally during the writing process in an environment of working memory (WM): transcription skills, oral language skills, and executive functions (EF). This model provides a framework for the study of children’s writing development, as it specifies the constraints that influence the writing process. In comparison to the preponderance of studies concerned with transcription skills and oral language skills, fewer studies have studied the importance of EF. Furthermore, it is postulated that the relative influence of each of these component skills on children’s written composition changes over time. Whereas transcription skills are considered critical at the early stage of writing development, automaticity with these skills frees up WM resources for implementing language skills and EF. These latter skills are thus expected to play a more constraining role in later written composition (Berninger & Swanson, 1994; Berninger & Winn, 2006). However, the majority of studies have focused on concurrent predictors of writing. Longitudinal predictive studies are lacking, particularly in the upper elementary grades when important steps in writing development are hypothesized to alter the interaction between the components. In light of these gaps in the literature, the present study assessed the predictive role of these component skills for the development of narrative writing in typically developing children in the upper elementary grades.

A first critical component of writing is transcription (Berninger & Winn, 2006). Transcription skills include handwriting fluency and word spelling (Berninger, 2000). These skills are essential for translating language representations into written symbols. In young writers, a lack of automaticity in transcription skills may largely constrain content generation and writing fluency, by increasing children’s processing load of their already limited WM resources (McCutchen, 1996). As such, cross-sectional research has found handwriting and spelling to be closely associated with written composition in elementary school, especially between kindergarten and the early elementary grades (e.g., Kim et al., 2011; Kim, Al Otaiba, Folsom, Gruelich, & Puranik, 2014; Puranik, Al Otaiba, Folsom, & Gruelich, 2012; Wagner et al., 2011). From a longitudinal perspective, Kent, Wanzek, Petscher, Al Otaiba, and Kim (2014) found that children’s spelling in kindergarten is predictive of their first grade writing, a result that was replicated by Kim, Al Otaiba, and Wanzek (2015) for third grade writing. A consistent longitudinal relationship between spelling and composing has also been reported for children ranging from first to seventh grade (Abbott, Berninger, & Fayol, 2010). Relatedly, instruction aimed at improving handwriting (e.g., Berninger et al., 1997; Graham, Harris, & Fink-Chorzempa, 2000) or spelling (e.g., Berninger et al., 1998) has been shown to improve composition in beginning writers.

The second component of writing, oral language skills (Berninger & Winn, 2006), equally constitutes an important cornerstone of the text generation process in writing. Oral language skills serve to translate ideas into language representations at the word, sentence, and discourse level. For instance, writers draw on their vocabulary knowledge to convey their ideas in writing and structure them into sentences (Berninger et al., 1992). The development of a rich and varied vocabulary can therefore be seen as an essential step in becoming a proficient writer (Baker, Gersten, & Graham, 2003; Roth, 2000). Also grammatical skills are considered to be important in text generation, as they enable the expression of complicated relationships among ideas (Coirier, 1996). A lack of adequate grammatical skills may impede sentence construction during writing, and result in shorter text, syntactically less complex sentences and a reduced compositional quality (Graham & Harris, 1989; Saddler & Graham, 2005; Tindal & Parker, 1989). Oral language skills have indeed been found to contribute concurrently to writing in children ranging from kindergarten to the intermediate grades of elementary school (Abbott & Berninger, 1993; Hooper, Roberts, Nelson, Zeisel, & Kasambira-Fannin, 2010; Kim et al., 2011; Kim, Al Otaiba, Folsom, Gruelich, & Puranik, 2013; Olinghouse, 2008; Olinghouse & Leaird, 2009). Further evidence of the role of oral language skills in writing comes from studies involving children with oral language impairment (Bishop & Clarkson, 2003; Dockrell, Lindsay, Connelly, & Mackie, 2007). In addition, the effectiveness of instruction in syntax for enhancing writing performance among elementary grade students underscores the significance of grammatical skills for writing development (Saddler, Behforooz, & Asaro, 2008; Saddler & Graham, 2005). Longitudinal evidence regarding the contribution of oral language skills to writing is limited. Coker (2006) found that receptive vocabulary proficiency in first grade was concurrently related to the quality and quantity of writing, but did not predict narrative writing growth from first to third grade. By contrast, Hooper et al. (2010) found that oral language skills prior to kindergarten entry predicted the rate of growth in narrative writing between third and fifth grade, suggesting that oral language skills only become predictive of writing in later grades.

The third component underlying writing performance is executive functioning. EF may either refer to the cognitive processes of planning, translating, reviewing, and revising that manage self-regulation of the writing process, or to the low-level EF that scaffold these high-level EF (Berninger & Richards, 2002, 2010). Low-level EF can be viewed as cognitive subcomponents of a single supervisory attentional mechanism, and typically include inhibition, updating of WM, and shifting (Miyake et al., 2000). Inhibition is characterized by abilities that include (1) selectively attending to specific stimuli while suppressing attention to other stimuli (*selective attention*), (2) staying on task and completing the task despite distractors (*sustained attention*), and (3) inhibiting prepotent responses (*response inhibition*). Updating of WM refers to the ability to keep relevant information, such as representational structures, in memory, mentally manipulate such information and act on account of it. Finally, shifting, also referred to as cognitive flexibility, includes the ability to quickly and flexibly adapt to changing situations, such as tasks or mental sets (Diamond, 2013). EF improve sequentially through childhood, aligning with growth spurts in the maturation of the frontal lobes (Anderson, 2002).

While empirical research has only recently started to unravel the role of these low-level EF in writing, they each bear a clear relevance to the complex process of writing. Inhibition may be engaged during planning to suppress knowledge that writers do not want to include in their composition. Similarly, while translating ideas into language, writers need to inhibit inappropriate lexical representations and syntactic structures, and select a relevant set of words and phrase structures (Kellogg, Whiteford, Turner, Cahill, & Mertens, 2013; Olive, 2011). Shifting, in turn, may support the translation process by constantly switching between subprocesses and knowledge (Quinlan, Loncke, Leijten, & Van Waes, 2012). Updating of WM, finally, may be involved in monitoring and integrating new information in WM, in order to sustain the writing process. More specifically, as composing progresses, the writer needs to update the contents of WM in line with the text produced so far (Olive, 2011; St Clair-Thompson & Gathercole, 2006).

The relative contribution of EF to writing in developing writers has received scant attention compared to the other two components, as it is generally assumed that young writers do not exhibit much self-initiated executive control during composition due to their immature transcription skills and limited capacity of WM (Bereiter & Scardamalia, 1987; McCutchen, 1988). While this may be true for the late developing high-level EF of planning (McCutchen, 1988; Scardamalia & Bereiter, 1986), recent empirical evidence suggests that low-level EF are involved in the early development of written language skills in elementary school (Altemeier, Abbott, & Berninger, 2008; Altemeier, Jones, Abbott, & Berninger, 2006; Berninger et al., 2006; Drijbooms, Groen, & Verhoeven, 2015; Hooper, Swartz, Wakely, de Kruif, & Montgomery, 2002; Hooper et al., 2011; Kent et al., 2014; Kim et al., 2013; Kim, Al Otaiba, & Wanzek, 2015; Thomson et al., 2005). However, few studies have used an extensive test battery of neuropsychological measures to study EF. Some have used a single latent construct summarizing EF measures (Hooper et al., 2011), whereas others have used a limited test battery to assess EF (Altemeier et al., 2006, 2008) or employed parents' and teachers' ratings of attentiveness (Kent et al., 2014; Kim et al., 2013, 2015; Thomson et al., 2005). Furthermore, while Kent et al. (2014) found attention regulation in kindergarten to be longitudinally predictive of both composition quality and fluency in first grade, other longitudinal studies have failed to replicate these findings for second (Hooper et al., 2011) and third grade writing outcomes (Kim et al., 2015). Moreover, longitudinal investigations tracing the predictive role of executive control beyond the early grades of elementary school are lacking. The importance of EF for writing in the upper elementary grades is, however, evident in the findings of intervention research, showing that training EF and attentional processes in fourth to sixth graders significantly improves children’s compositional skills (Chenault, Thomson, Abbott, & Berninger, 2006) and spelling performance (Hooper, Wakely, de Kruif, & de Schwartz, 2006).

Taken together, while research investigating the component skills involved in writing has grown substantially, the majority of studies focus on concurrent predictors, with a particular emphasis on transcription and oral language skills. Longitudinal predictive studies, that also include an extensive array of EF, are lacking, with the upper elementary grades being a particularly understudied age range. These grades are, however, a critical period to examine the predictive role of these components, as the nature of writing and writing tasks changes substantially from fourth grade onwards. With transcription skills becoming automatized after fourth grade and beyond, more cognitive resources should become available for implementing oral language skills and EF (Berninger & Swanson, 1994; Berninger & Winn, 2006). Simultaneously, from fourth grade onwards, task requirements in the curriculum change and the translation process becomes more complex (Altemeier et al., 2008; Berninger & Chanquoy, 2012; Mehta, Foorman, Branun-Martin, & Taylor, 2005). Whereas writing in the early grades involves primarily learning to write letters, spell, and compose short texts, by fourth grade children are expected to compose increasingly complex and lengthy written texts, requiring closer attention to the structural and compositional aspects of the text as a whole (Berninger, Abbott, Whitaker, Sylvester, & Nolen, 1995; Graham, Harris, & Olinghouse, 2007; Wagner et al., 2011). As such, children are required to call upon sophisticated lexical and syntactic skills for the translation of ideas into coherent, extended discourse (Nippold, 2004; Shanahan, 2006). With such complex tasks, children have to engage more extensively in self-regulation and attentional control in order to manage the increasingly complex translation of ideas into language, the complex writing environment, the constraints imposed by the topic, and the associated compositional processes, such as revising, editing, organizing, and planning written expression (Altemeier et al., 2008; Hooper et al., 1993; Kellogg, 1987; Scardamalia & Bereiter, 1986). Hence, in such a context EF presumably become more critical to writing quality.

An increasingly popular approach to the assessment of writing quality is through the analysis of linguistic features. Frequently recurring features include measures of productivity (e.g., text length), complexity (e.g., syntactic complexity), and macro-organization (e.g., structure or content; Puranik, Lombardino, & Altmann, 2008; Wagner et al., 2011). Unlike holistic ratings, these features concern characteristics that can be quantitatively measured (Crossley, Weston, McLain Sullivan, & McNamara, 2011). While many studies have focused on one specific feature as a proxy for text quality, multi-feature studies that longitudinally examine the development of these features within the same group of children are much less common (e.g., Kent et al., 2014; Kim et al., 2015). Yet, it appears that multi-feature approaches are better able to reflect the multidimensional nature of written composition. Particularly, there is growing evidence that the three measures just mentioned correspond to three relatively independent and dissociable dimensions of writing. Hence, individual children have been shown to vary in their writing performance at each of these dimensions (e.g., Kim et al., 2015; Puranik et al., 2008; Wagner et al., 2011). While the validity of these measures for writing assessment has been established, research still has to validate which measures are most sensitive to capture growth in children’s writing (Puranik, Wagner, Kim, & Lopez, 2012).

## The present study

The present study aimed to complement existing understanding of the role of component skills in writing development in two respects. First, we examined the longitudinal, rather than concurrent, predictive role of these skills. Second, we focused on writing growth in the upper elementary grades, given the substantial changes in writing development generally occurring in this age range. In particular, this study aimed to investigate how transcription skills, oral language skills, and EF as assessed by a broad neuropsychological test battery, predict narrative writing growth over time from fourth to sixth grade. Narrative writing was chosen because it marks the transition from writing letters and short texts to more substantial writing. Narratives are an integral part of educational curricula from the early primary grades throughout high school (Roth, 2000), and are among the most common writing assignments in elementary school (Cutler & Graham, 2008). Moreover, there is a substantial body of research demonstrating a link between children’s narrative abilities and their academic success (for a review, see Boudreau, 2008).

In order to monitor growth in narrative writing, children’s written compositions were evaluated on three measures: text length, syntactic complexity, and story content. Given this study design, specific research questions were as follows:

1. To what extent do measures of text length, syntactic complexity, and story content of narrative composition develop between fourth and sixth grade?
2. To what extent are the measures of text length, syntactic complexity, and story content longitudinally predictive of later measures, within and across themselves?
3. To what extent do transcription skills, oral language skills, and EF predict growth in these measures of narrative writing?

It was expected that each measure would show development over time, though the magnitude of progression may differ between measures. Further, in accordance with the relative independence of the different dimensions of writing, it was hypothesized that the measures would be longitudinally predictive within but not across themselves, confirming their nature as dissociable dimensions of writing. Finally, based on the changes that characterize later writing development, we predicted that oral language skills and EF would be powerful predictors of later narrative writing, whereas transcription skills would constitute a relatively less important predictor of growth in this age group.

## Method

### Participants

Participants were recruited from four mainstream elementary schools in the Netherlands, with on average a middle to middle-high socio-economic background according to the Netherlands Institute for Social Research. Children displaying learning or behavioral problems were excluded from participation. Upon initial measurement in fourth grade, the sample consisted of 102 children (age *M* = 9.6 years, *SD* = 5.74 months; 46.1 % girls). This sample has been reported upon in an earlier study (Drijbooms, Groen, & Verhoeven, 2015). Due to dropouts throughout the years, the final sample comprised 93 children (46.6 % girls) in the second testing phase in sixth grade (age *M* = 11.1 years, *SD* = 5.29 months). Analyses were conducted on the data of children who participated both in fourth and in sixth grade. Active parental consent was obtained for each child. All participating children spoke Dutch and were raised in the Netherlands. Seven percent of the children were bilingual as they also spoke an additional language at home. However, the bilingual children did not perform significantly worse on vocabulary, oral grammar, and spelling than the monolingual children, so their data were retained for the analyses.

### Procedure

In fourth grade, children’s transcription skills, oral language skills, EF, and narrative writing skills were assessed. Assessments for handwriting fluency, oral language skills, and EF were individually administered in two administration blocks (block A and block B) by the first author and two trained research assistants. Block A consisted of the oral language measures, and the measure of handwriting fluency. Block B comprised the EF measures. Administration of the blocks was counterbalanced to minimize order effects. The narrative writing skills and spelling skills were group-administered by the first author. In sixth grade, only narrative writing skills were re-administered.

### Measures

#### Narrative writing

To assess children’s narrative writing skills, a picture-elicitation task—the Expression, Reception and Recall of Narrative Instrument (ERRNI; Bishop, 2004)—was administered. The ERRNI is suitable for all age groups, ranging from 6 years until adulthood. Although the instrument was originally designed for assessing oral narrative skills, the ERRNI procedures have previously been successfully adapted for assessing written narrative skills (Cragg & Nation, 2006). The instrument includes two parallel tasks, the Beach Story and the Fish Story, that are each composed of a sequenced story of 15 pictures. To elicit a written narrative, children were each presented with the picture booklet for the Fish Story. They were allowed to consult the pictures throughout the composition task. Children were instructed to look carefully at the pictures, before starting to write. The duration of the task and the length of the narrative were not imposed. The same story task was used in fourth and in sixth grade, in order to control the content and to monitor children’s growth in narrative writing through a direct and straightforward comparison of measures in fourth and in sixth grade. All narratives were transcribed using Computerized Language ANalysis program (CLAN) from Child Language Data Exchange System (CHILDES; MacWhinney, 2000).

Following the coding scheme developed by Puranik, Lombardino, and Altmann (2007, 2008), three measures were derived from children’s written narratives: text length measured by the total number of words, syntactic complexity measured by the mean length of a t-unit in words, and story content measured by the total number of ideas. Total number of words is a frequently used measure of compositional fluency and productivity, and a strong predictor of writing quality. The mean length of a t-unit in words was calculated by dividing the number of words produced by the number of t-units. A t-unit, or minimal terminable syntactic unit, is defined as an independent main clause, with any subordinate clauses associated with it (Hunt, 1965; Loban, 1976). Total number of ideas was calculated according to standard ERRNI procedures. The instrument contains a list of 24 main ideas that are represented in the story. Two points were awarded for each idea included in the narrative, one point was given when the idea was represented only partially. For example, for the main idea “The boy waves goodbye and goes home”, a child was credited with one point only if the waving was included without mentioning the boy going home, or vice versa. To be credited with two points, reference also had to be stated clearly. For example, for the main idea “The boy and the girl buy an ice cream”, stating that “they buy an ice cream” was only credited with one point when reference was ambiguous or did not result from the previous linguistic context. Two raters scored the story content of 20 % of the transcripts in common to practice the scoring scheme. Disagreements were resolved through discussion. Afterwards, half of the transcripts were scored by the first rater and half by the second rater. Twenty percent of the transcripts was scored by both raters to determine inter-rater reliability. The inter-rater reliability was calculated as .92.

#### Transcription skills

Children’s handwriting fluency and spelling skills were both assessed. Handwriting fluency was assessed by means of the Systematic Screening of Handwriting Difficulties (Van Waelvelde, De Mey, & Smits-Engelsman, 2008), requiring children to copy a short text during 5 min. Handwriting fluency is calculated by counting the number of letters written in 5 min. Test–retest reliability is reported to be .69 (Van Waelvelde, Hellinckz, Peersman, & Smits-Engelsman, 2012).

Spelling skills were assessed through a standardized Dutch dictation task, the “PI-dictee” (Geelhoed & Reitsma, 1999), containing 135 words that gradually increase in difficulty. Words were presented in sentences, after which children were instructed to write down the repeated word from each sentence. The raw score was the number of words spelled correctly (max. score = 135). Test–retest reliability for this task is reported as .91 (Geelhoed & Reitsma, 1999).

#### Oral language skills

Oral language skills were assessed by measures of receptive vocabulary knowledge and oral grammar. Vocabulary knowledge was measured through the Peabody Picture Vocabulary Test (PPVT-III-NL; Dunn & Dunn, 2005). Children were shown a test page containing four pictures and were asked to indicate the target picture that corresponded best to the word presented orally by the experimenter. Words were presented in a pre-determined block of 12 trials, and testing was discontinued when the child missed eight or more items in a 12-item set. Raw scores (max. = 204) were used in the analyses. Internal consistency reliability is reported to be .95 (Dunn & Dunn, 2005).

Oral grammar was assessed by measuring the mean length of a t-unit in words as an index of syntactic complexity during an oral narrative production task. The Beach Story of the ERRNI was used to elicit the oral narrative (Bishop, 2004). The mean length of a t-unit is automatically calculated by CLAN, and therefore does not require a reliability estimate. While the same type of measure (mean length of a t-unit) was thus collected to assess oral grammatical skills and syntactic complexity of narrative writing, different stories were used to obtain the measures in the two different modalities. This measure of oral grammar was preferred over a measure resulting from a more traditional sentence repetition task (e.g., Verhoeven & Vermeer, 2001), because it tends to be more sensitive to capture grammatical ability in older children (Vender et al., 1981), and avoids confounds such as the involvement of verbal working memory, thereby reducing the risk of under- or overestimating children’s grammatical skills (e.g., Adams & Gathercole, 2000).

#### Executive functions

For the domain of EF, a battery of tasks was chosen to represent the three core low-level EF of inhibition, updating, and shifting, and the high-level EF of planning. Multiple tasks were chosen in order to represent all facets of the EF. To assess inhibition four tasks were selected: the subtest Sky Search of the Test of Everyday Attention for Children (Tea-Ch Sky Search; Manly, Robertson, Anderson, & Nimmo-Smith, 1999) was administered to assess *selective attention*. This subtest includes an A3-sheet which is full of spaceships that fly in pairs. Children were instructed to circle as many pairs of identical spaceships, as quickly as possible. To control for motor speed, a motor control version of the task, during which children have to mark pairs of spaceships on a separate A3-sheet that only displays identical pairs, was subsequently administered. The total time in seconds needed to complete the motor control version was subtracted from the total time needed to complete the experimental task, yielding a selective attention score. Test–retest reliability for this task is reported as .80 (Manly et al., 1999). To obtain a measure of *sustained attention*, the Letter Digit Substitution Task (LDST; Jolles, Houx, Van Boxtel, & Ponds, 1995) was administered. Children were given a sheet with a key on top of the page, which paired nine letters with nine digits, and the test items, more particularly letters, printed beneath the key. Children were then required to write the corresponding digits below each letter, as indicated by the key. They were instructed to substitute as many letters as possible within the test time of 90 s. The number of correct substitutions made in 90 s was used as the raw score. Test–retest reliability for this task is reported as .88 (Jolles et al., 1995). Two tasks, the subtest Walk Don’t Walk (Tea-Ch Walk Don’t Walk) and the subtest Opposite Worlds (Tea-Ch Opposite Worlds) of the Tea-Ch, were used to assess *response inhibition*. Walk Don’t Walk is a subtest in which children had to track auditory sounds (Go-sounds) on a sheet by marking footprints on an A4 sheet, and were required to stop marking once a target sound (No-Go sound) was presented. No-Go sounds occurred at random, unpredictable intervals. The total number of correct responses out of 20 was taken as the raw score for this task. The test–retest reliability for this task is reported as .71 (Manly et al., 1999). The Opposite Worlds subtest measures verbal response inhibition, and required children to say the opposite of a logical response. In the same world condition, children were asked to name, as quickly as possible, the digits 1 and 2 that were scattered along a path. In the opposite world condition, children were required to say two for the digit one and one for the digit two. The raw score was calculated as the time in seconds needed to complete the opposite world condition. Test–retest reliability for this task is reported as .85 (Manly et al., 1999). To assess updating skills, the Wechsler Intelligence Scale for Children-IV-Integrated Digit Span subtest (WISC-IV-I Digit Span; Wechsler, 2004), including a Forward Digit Span condition and a Backward Digit Span condition, was administered. The Forward Digit Span required children to repeat a string of digits in the right order. The first trial started with two digits, and increased with one digit after a level had been presented twice. For each correctly recalled trial, children were given one point. In the Backward Digit Span, the procedure was similar, but children were asked to repeat the digits in the reverse order. The total raw score for this task was calculated by adding the raw score of the Forward Digit Span (max. = 14) and the raw score of the Backward Digit Span (max. = 14). The internal consistency reliability for this task was calculated as .78. Furthermore, two shifting tasks were chosen: the Letter Fluency subtest from the Delis–Kaplan Executive Function System (D-KEFS-Letter Fluency; Delis, Kaplan, & Kramer, 2001) was used to tap phonemic verbal fluency, which requires mentally shifting between multiple subsets of words. Children were asked to generate as many words as possible starting with the letter M, and with the letter K, with 60 s allowed for each letter. The raw score was calculated by adding the number of correct words for both letters. Test–retest reliability for this task is reported as .76 (Korkman et al., 1998). The Trail Making Test from the D-KEFS (D-KEFS-TMT; Delis et al., 2001) was administered to assess cognitive flexibility. During this task, children were asked to connect numbers (1–16) and letters (A–P) in an ascending order by drawing lines, with the additional challenge of alternating between the numbers and letters (1-A-2-B-3-C, etc.). Children were instructed to complete the task as fast and as accurately as possible. The raw score was the time in seconds needed to complete this task. Test–retest reliability for this task is reported as .89 (Delis et al., 2001). Finally, the high-level EF of planning was assessed by means of the Tower of London (TOL; Shallice, 1982). The task required children to move five discs across three pegs, from a prearranged initial position to a goal position, as indicated by a picture. Children were instructed to achieve the goal position in as few moves as possible while adhering to the following rules: (1) move only one disk at a time, (2) never place larger discs on smaller discs, and (3) use only one hand while moving discs. Scores were assigned according to the number of moves needed to achieve the different goal positions. The total raw score was obtained by adding the score of each goal position (max. = 30). Internal consistency reliability for this task is reported as .84 (Delis et al., 2001).

To reduce and summarize the data, a principal component analysis with varimax rotation, described in detail in Drijbooms et al. (2015), was run on all the EF measures. Three factors were found (Eigen values: 2.32, 1.09, and 1.03). The first factor showed high loadings on Tea-Ch Walk Don’t Walk (.67), Tea-Ch Opposite Worlds (.83), LDST (.56), and D-KEFS-TMT (.56). The second factor showed high loadings on WISC-IV-I Digit Span (.49), D-KEFS-Letter Fluency (.50), and Tea-Ch Sky Search (.83). The third factor, finally, showed high loadings on D-KEFS-TMT (.52), and TOL (.91). Given these results, the EF measures were consolidated into three factors, labeled Inhibition, Updating, and Planning respectively. Shifting was thus not distinguished as a separate factor. This could be explained by recent evidence suggesting that in children shifting may not be dissociable from inhibition and updating (Hughes, Ensor, Wilson, & Graham, 2009; St Clair-Thompson & Gathercole, 2006; Wiebe et al., 2011), but builds highly upon them (van der Ven, Kroesbergen, Boom, & Leseman, 2013), and emerges later in development (Diamond, 2013). Although the factors do not entirely correspond to the fractionation of EF as put forward by the literature, they do reflect a distinction between low-level EF (inhibition and updating) and high-level EF (planning). The factor scores were used as variables in the analyses.

## Results

Preliminary analyses included descriptive statistics (see Table 1) and correlational analyses (see Table 2). A number of patterns are evident in the correlations. First, the predictor variables and initial writing measures were differentially correlated with later writing measures. For later text length, only a correlation with initial text length could be established. For later syntactic complexity, significant correlations with all measures were found, except with handwriting fluency, spelling, vocabulary, and updating skills. For later story content, a significant correlation was established with initial text length, initial story content, oral grammar, and later text length. Second, individual differences on each narrative measure were consistently correlated longitudinally between fourth and sixth grade. The magnitude of the autoregressive paths was moderate and similar for each measure (range = .30–.39).

**Table 1:** Descriptive statistics for the measures of the written narratives, transcription skills, oral language skills, and executive functions

*n* = 93	Fourth grade		Sixth grade	
	Mean (*SD*)	Min–max	Mean (*SD*)	Min–max
The written narratives				
Text length	240.61 (104.95)	75–560	236.78 (99.97)	70–538
Syntactic complexity	6.35 (1.42)	2.68–10.27	7.69 (1.53)	4–10.94
Story content	26.49 (6.00)	12–40	26.34 (5.57)	12–40
Transcription skills				
Handwriting fluency	177.08 (39.65)	63–260		
Spelling	95.31 (16.61)	41–127		
Oral language skills				
Oral grammar	7.61 (1.35)	4.86–10.66		
Vocabulary	115.59 (9.43)	96–141		
Executive functions				
Tea-Ch Sky Search	4.40 (1.61)	2–12.90		
Tea-Ch Walk Don’t Walk	14.06 (3.26)	3–20		
Tea-Ch Opposite Worlds	31.47 (5.13)	22–47		
LDST	32.96 (7.28)	14–49		
WISC-IV-I digit span	12.01 (2.30)	5–20		
D-KEFS-letter fluency	14.65 (4.40)	4–28		
D-KEFS-TMT	113.04 (40.53)	38–240		
TOL	15.14 (2.72)	6–21		

*Tea*-*Ch* test of everyday attention for children, *LDST* letter digit substitution task, *WISC*-*IV*-*I Digit Span* Wechsler Intelligence Scale for children-IV-integrated digit span, *D*-*KEFS*-*Letter Fluency* Delis–Kaplan executive function system letter fluency, *D*-*KEFS*-*TMT* Delis–Kaplan executive function system trail making test, *TOL* tower of London

**Table 2:** Correlations between measures of the written narratives in fourth grade, measures of the written narratives in sixth grade, transcription skills, oral language skills, and executive functions

	1	2	3	4	5	6	7	8	9	10	11	12	13
Fourth grade measures													
1. Text length	1												
2. Syntactic complexity	.35**	1											
3. Story content	.52**	.45**	1										
4. Handwriting fluency	.31**	.23*	.32**	1									
5. Spelling	.29**	.22*	.16	.22*	1								
6. Grammar	.17	.44**	.26*	.06	.15	1							
7. Vocabulary	.17	.02	.24*	.10	.13	−.09	1						
8. Inhibition	.27**	.23*	.17	.24*	.26*	.03	.02	1					
9. Updating	.28**	.04	.20	.17	.12	.11	.05	−.03	1				
10. Planning	−.01	.05	.12	.05	.01	.10	.06	.02	−.03	1			
Sixth grade measures													
11. Text length	.30**	.16	.15	.16	.10	.14	.00	.17	.08	.03	1		
12. Syntactic complexity	.22*	.39**	.23*	.10	.05	.37**	−.00	.25**	−.07	.22*	.24*	1	
13. Story content	.35**	.15	.37**	.16	.09	.23*	.15	.20	.06	.06	.23*	.13	1

* *p* < .05; ** *p* < .01

In order to answer the first research question, to what extent do text length, syntactic complexity, and story content develop between fourth and sixth grade, three paired sample *t* tests were conducted for each of the writing measures. The results evidenced a significant increase in syntactic complexity, *t*(92) = 7.91, *p* < .001, *d* = .82, but no developmental progression was observed for text length, *t*(92) = .31, *p* = .76, *d* = .03, nor for story content, *t*(92) = .22, *p* = .82, *d* = .02.

With regard to the second and third research question, a series of path analyses were conducted with AMOS 22 (Arbuckle, 2013), using maximum likelihood estimation method. Non-significant paths (i.e. paths exceeding the *p*-level of <.05) were removed stepwise to obtain the most parsimonious models. The fit of the models was evaluated using the following fit indices: a model fits well if the Chi square (*χ* ^2^) exceeds .05 (Ullman, 2001), the goodness of fit index (GFI), the comparative fit index (CFI), the adjusted goodness of a fit (AGFI) and the normed fit index (NFI) are greater than .90 and the root mean square error of approximation (RMSEA) is lower than .08 (Hu & Bentler, 1999).

In order to answer the second research question, to what extent are the initial measures of text length, syntactic complexity, and story content longitudinally predictive of later narrative measures, a simplex autoregressive and cross-lagged model was constructed to test how each measure influences itself over time (within-measure autoregressive longitudinal path) and how each measure crosses over to influence another measure at a subsequent time (between-measures longitudinal cross-lagged path). Hence, this model was evaluated to determine (a) the degree of stability of each measure over time, and (b) the longitudinal relationships across the measures. As no developmental progression was observed for text length or for story content, the only cross-lagged paths included were from text length in fourth grade to syntactic complexity in sixth grade, and from story content in fourth grade to syntactic complexity in sixth grade. Neither of the two cross-lagged paths turned out to be significant, indicating that text length and story content in fourth grade were not predictive of syntactic complexity in sixth grade. Hence, the best fit for the model was obtained when the non-significant cross-lagged paths were removed: *χ* ^2^ (6) = 5.32, *p* = .50, GFI = .98, CFI = 1.00, AGFI = .94, NFI = .95, RMSEA = .00. An examination of the values of the autoregressive path coefficients revealed that each measure in fourth grade had a significant longitudinal path and explained unique variance in itself in sixth grade (text length: standardized coefficient = .27; syntactic complexity: standardized coefficient = .38; story content: standardized coefficient = .37). Hence, the longitudinal relationships within measures reflect that each of these measures is relatively stable across the upper elementary grades. Furthermore, the lack of a longitudinal relationship across the measures confirms that each measure constitutes a relatively independent and dissociable dimension of writing.

**Fig. 1:**
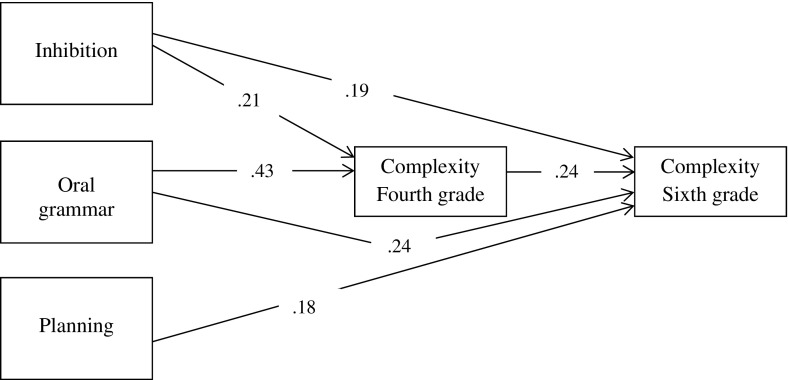
Path model with EF and oral grammar in fourth grade, and syntactic complexity of narratives in fourth and in sixth grade. All path coefficients are significant, *p* < .05. *Note:* Paths between vocabulary and transcription skills in fourth grade and syntactic complexity in fourth and in sixth grade were estimated, but were not found to be significant

In order to answer the third research question, to what extent do component skills predict growth in each measure of narrative writing, a second path-model was constructed. Considering the lack of developmental progression for text length and story content, and the longitudinal independence of the measures, we decided to construct a path-model that only considered the contribution of transcription skills, oral language skills and EF to development in syntactic complexity. First, a saturated model was fitted to the data with all possible paths from the predictor variables to the outcome variable. Non-significant paths were then dropped iteratively from the model, examining changes in fit, resulting in the final model as depicted in Fig. 1. This model had a strong fit: *χ* ^2^ (4) = .98, *p* = .91, GFI = 1.00, CFI = 1.00, AGFI = .98, NFI = .98, RMSEA = .00. The path model showed that syntactic complexity in sixth grade was, in addition to the stability effect of syntactic complexity in fourth grade (*β* = .24), predicted by inhibition (*β* = .19), planning (*β* = .18), and oral grammar (*β* = .24). Inhibition and oral grammar also indirectly influenced syntactic complexity in sixth grade through their concurrent contribution to syntactic complexity in fourth grade (respectively: *β* = .21, and *β* = .43).

## Discussion

In the present study, we investigated development of narrative writing in the upper elementary grades, and its predictors, by assessing narrative writing along three dimensions and simultaneously administering a large test battery of transcription skills, oral language skills, and EF. In answer to our first and second research questions, we found that syntactic complexity, but not text length nor story content, improved significantly with age, that each measure was longitudinally predictive within itself, and that there were no longitudinal relationships across measures. Regarding the third research question, we focused exclusively on the longitudinal predictors of syntactic complexity, in the absence of a developmental progression in text length and story content. Findings showed that oral grammar and the EF of inhibition and planning were longitudinally related to syntactic complexity of written narratives. Our results can be interpreted in light of the changes in the nature of writing and writing tasks that characterize writing development in the upper elementary grades.

The developmental progression of syntactic complexity between fourth and sixth grade is convergent with the general idea that syntactic coding is an increasingly important factor in writing proficiency from fourth grade onwards until college (e.g., Berninger et al., 2010). It confirms that development of writing involves the use of increasingly complex syntax, a view which has been advocated in seminal work by Hunt (1965) and Loban (1976), and which has found support in several subsequent studies (e.g., Beers & Nagy, 2009; Berninger, Nagy, & Beers, 2011). More particularly, development in syntactic complexity is a key feature of later language development (Nippold, 2007) and reflects children’s growing ability to express complex ideas, and their frequent exposure and familiarity with the literate genre (Fang, Schleppegrell, & Cox, 2006). In this sense, children’s progress on syntactic complexity is fully commensurate with the more complex writing tasks that children are exposed to in the upper elementary grades (e.g., Berninger et al., 1995; Wagner et al., 2011). It is somewhat surprising that text length and story content did not increase with age, as they represent two widely examined dimensions of writing, that is productivity and macro-organization respectively. The lack of ceiling effects and the standard variation of our sample support the idea that the task itself theoretically provides enough possibilities for growth. A tentative explanation for the lack of a developmental increase could perhaps be found in motivational constraints related to the nature of the task. The relatively simple picture-description task may not have been challenging enough for the sixth graders, thereby constraining children’s motivation to perform well on the task (Troia, 2011). Syntactic complexity may be less affected by motivational constraints. More specifically, for young writers syntax is still a part of implicit linguistic knowledge that is applied unconsciously, rather than explicitly and intentionally (Ravid & Tolchinsky, 2002). In agreement with previous studies (e.g., Kim et al., 2014, 2015; Puranik et al., 2008; Wagner et al., 2011), this developmental finding, along with the longitudinal relationships within but not across measures, underscores the idea that writing is not a single dimension but is composed of multiple dimensions that may each show a different developmental trajectory, and may be differentially subject to writing constraints. While it is beyond the scope of the current study to fully evaluate the dimensionality of writing, these findings do emphasize the importance of assessing writing at different dimensions.

Regarding the longitudinal predictors of syntactic complexity, the findings of the present study showed how oral grammar and EF, but not transcription skills, in fourth grade relate to later syntactic complexity of narrative writing. This confirms our hypothesis and supports a developmental theory of writing (Berninger & Swanson, 1994; Berninger & Winn, 2006). Prior research has found transcription skills, and particularly spelling, to be longitudinally predictive of writing skills in the early and middle grades of elementary school (e.g., Abbott et al., 2010; Kent et al., 2014; Kim et al., 2015). Examining more specifically the dimension of complexity, Wagner et al. (2011) found a significant concurrent relationship between handwriting fluency and syntactic complexity of written composition for first, but not for fourth graders. Similarly, in a study by Kim et al. (2014), spelling was a unique predictor of syntactic complexity of first graders’ narrative written composition. Together with these previous findings, the lack of a longitudinal relationship of transcription skills to syntactic complexity in the present study indicates that their influence declines once children get older and their handwriting and spelling skills become automatized (Berninger & Swanson, 1994). Hence, the ability to produce syntactically complex sentences at this stage of writing development seems to be no longer constrained by handwriting and spelling skills. This should imply increased availability of cognitive resources for higher-order processes, such that it allows children to employ their accumulated language and EF skills to produce text (Berninger & Winn, 2006).

Indeed, the longitudinal contribution of oral language skills to syntactic complexity confirms that proficiency in spoken sentence production in fourth grade boosts the development of the ability to write syntactically complex sentences. More specifically, children with superior oral sentence construction skills may have access to a larger syntactic repertoire, which facilitates written sentence production. This extends previous research with younger children (e.g., Abbott & Berninger, 1993; Kim et al., 2013, 2015; Olinghouse, 2008), by revealing that oral language sophistication is longitudinally related to a specific dimension of narrative writing. It is interesting to note that, although we used the same kind of measure, i.e. mean length of a t-unit during a narrative production task, to measure oral grammar on the one hand and syntactic complexity of narrative writing on the other hand, the two were only moderately correlated. This demonstrates that written language is not simply spoken language written down (Bereiter, 1980), and that development in syntactic complexity of narrative writing is influenced by factors other than oral language ability as well. While receptive vocabulary was not predictive of syntactic complexity in the current study, this not necessarily implies that vocabulary is not important for written composition. Rather, we hypothesize that our vocabulary measure might be more sensitive for capturing individual differences in other dimensions of writing, such as macro-organization.

Importantly, besides oral language skills, we found that executive control contributes to development in syntactic complexity. More particularly, children who exhibited higher planning and inhibition skills in fourth grade were more likely to improve on syntactic complexity of their narratives between fourth and sixth grade. Executive functions of planning and inhibition may enhance the syntactic complexity of narratives through an increased ability to channel resources to specific problems that occur during writing. More specifically, a writer who is writing down a syntactically complex sentence is more likely to be successful in doing so, if he is able to approach the writing task and its subtasks in a goal-oriented way, and inhibit immediate responses to other problems such as typological errors (Quinlan et al., 2012). Moreover, in producing sentences, a writer has to linguistically translate a preverbal semantic message into a grammatical structure (Alamargot & Chanquoy, 2001; Levelt, 1989). This process consists of drafting a syntactic and lexical plan, taking into account that the unordered elements of the preverbal message must end up in a linear and unidimensional sequence of words (Levelt, 1989). Determining the order of elements is thus a critical part of the production of a sentence, which requires considerable planning skills. Sentence production in this sense also requires keeping several alternative grammatical options in WM, and inhibiting irrelevant ones (Thornton & Light, 2006). While this explains the longitudinal contribution of inhibition to syntactic complexity, it leaves the question unanswered as to why the EF of updating WM did not contribute to the syntactic complexity of children’s written narratives. A possible explanation is that the syntactic structures used by the children do not place a high cognitive load on WM, because they are planned locally and incrementally instead of prior to writing (Nottbusch, 2010).

Overall, the predictive role of planning and inhibition confirms that EF are required for managing the production of complex texts (Graham & Harris, 2000; Kellogg, 1987; Scardamalia & Bereiter, 1986). Planning, in contrast to inhibition, plays only a longitudinal, but not a concurrent, predictive role. This seems to suggest that planning skills are not yet fully operational in fourth grade. Generally, developing writers have indeed been found to show little, overt planning behavior during composition, as their writing is constrained by non-automatic lower-level writing processes (McCutchen, 1988; Scardamalia & Bereiter, 1986).

From a theoretical perspective, the critical importance of EF for writing in the upper elementary grades as evidenced by this study confirms predictions of developmental models of writing. It further demonstrates that research investigating predictors of writing skills in children should include neuropsychological measures of EF. More broadly, this study enhances our current understanding of EF in writing, by specifying its contribution to a specific dimension of writing. Whereas the act of writing is frequently documented as a problem-solving activity, which requires executive functioning to manage complex cognitive processing, few writing studies have attempted to relate EF to specific aspects of the translation process of writing. From an educational perspective, the present study offers perspectives for instruction and assessment practices. Instructionally, our results imply that in order to improve children’s sentence production in written composition in the upper elementary grades, attention needs to be paid to enhancing children’s EF, and particularly planning and inhibition skills. This is aligned with the idea that children have to be trained more extensively in self-regulation skills in order to manage written composition (Altemeier et al., 2008; Graham & Harris, 2000). Such training may be crucial, as evidence exists that enhancing syntactic complexity of written composition positively impacts on overall compositional quality (Saddler & Graham, 2005; Saddler et al., 2008). Furthermore, although there is a general lack of syntax-focused instruction in current writing curricula (Beers & Nagy, 2009), our findings demonstrate that children do progress significantly on the syntactic complexity of written narratives in the upper elementary grades. Hence, syntactic complexity could be considered an important developmental marker of written language, and thus a sensitive indicator to monitor children’s progress in writing.

Some limitations of the present study should be acknowledged and point to directions for future research. First, although using written picture description tasks has several advantages, motivational constraints related to the task might have affected our results. Using more authentic narrative tasks, such as personal narratives, which children are often asked to engage in, both in and outside of school contexts, may help to overcome these motivational barriers. Moreover, despite the central role of narrative writing in the transition into more extended writing, it might not be fully representative of the complex writing tasks in the later grades of elementary school. More particularly, narrative writing does not cover writing-to-learn activities, during which writing is used as a tool to facilitate classroom learning and construct new knowledge (e.g., Bangert-Drowns, Hurley, & Wilkinson, 2004). Such writing activities are increasingly used beyond fourth grade, and pose higher cognitive and linguistic demands on the writer. Future studies with cognitively more challenging tasks and genres, such as for instance expository writing, are needed to determine how component skills contribute to different writing outcomes. Furthermore, while this study included the major component skills of writing according to developmental models of writing, several other potential predictors of writing have not been explored, such as reading skills, motivation, and instructional quality. Finally, monitoring the predictors of writing development over a longer time span, including both younger and older writers, could complete the picture of the changing relationships between component skills and writing across development.

In summary, the results of the present study support oral language skills and particularly EF as building blocks of writing development in the upper elementary grades. To our knowledge, this is the first study that confirmed such a longitudinal relationship for the complexity dimension of narrative writing. While further research into the multiple influences on writing is clearly warranted, the findings of the current study have provided initial, valuable information about the complex foundations of writing development in the upper elementary grades.
